# Glutathione-the “master” antioxidant in the regulation of resistant and susceptible host-plant virus-interaction

**DOI:** 10.3389/fpls.2024.1373801

**Published:** 2024-03-12

**Authors:** Edmund Kozieł, Katarzyna Otulak-Kozieł, Piotr Rusin

**Affiliations:** Institute of Biology, Department of Botany, Warsaw University of Life Sciences, Warsaw, Poland

**Keywords:** plant virus, glutathione metabolism, resistance response, plant defense, susceptible reaction

## Abstract

The interaction between plant hosts and plant viruses is a very unique and complex process, relying on dynamically modulated intercellular redox states and the generation of reactive oxygen species (ROS). Plants strive to precisely control this state during biotic stress, as optimal redox levels enable proper induction of defense mechanisms against plant viruses. One of the crucial elements of ROS regulation and redox state is the production of metabolites, such as glutathione, or the activation of glutathione-associated enzymes. Both of these elements play a role in limiting the degree of potential oxidative damage in plant cells. While the role of glutathione and specific enzymes is well understood in other types of abiotic and biotic stresses, particularly those associated with bacteria or fungi, recent advances in research have highlighted the significance of glutathione modulation and mutations in genes encoding glutathione-associated enzymes in triggering immunity or susceptibility against plant viruses. Apparently, glutathione-associated genes are involved in precisely controlling and protecting host cells from damage caused by ROS during viral infections, playing a crucial role in the host’s response. In this review, we aim to outline the significant improvements made in research on plant viruses and glutathione, specifically in the context of their involvement in susceptible and resistant responses, as well as changes in the localization of glutathione. Analyses of essential glutathione-associated enzymes in susceptible and resistant responses have demonstrated that the levels of enzymatic activity or the absence of specific enzymes can impact the spread of the virus and activate host-induced defense mechanisms. This contributes to the complex network of the plant immune system. Although investigations of glutathione during the plant-virus interplay remain a challenge, the use of novel tools and approaches to explore its role will significantly contribute to our knowledge in the field.

## 1 Introduction

The plant organisms, being generally static land organisms, are consistently exposed to a wide range of pathogens that are an ongoing danger to them. Consequently, plants have developed a sophisticated network of defense systems to protect themselves from pathogen incursions and the development of diseases (Ramirez-Prado et al., 2018; Saijo and Loo, 2020; Zechmann, 2020). The components of the plant defense system encompass physical changes in host cells, also known as constitutive defenses, such as the thickening of cell walls to stop external invaders (Martínez-González et al., 2018; Zhu and Li, 2021) or to hinder the translocation of pathogens, such as viruses, within the plant (Otulak-Kozieł et al., 2018a, b, 2020). However, physical barriers alone are often not sufficient to block pathogenesis. Therefore, plants activate internal chemical and molecular pathways to induce defense mechanisms and eliminate pathogenic invasions (Zogli and Libault, 2017; Gimenez et al., 2018; Ramirez-Prado et al., 2018; Hammerbach et al., 2019; Yang et al., 2019; Saijo and Loo, 2020; Zechmann, 2020). The speed and effectiveness of this response play a crucial role in determining the future fate of the plant host (Mandadi and Scholthof, 2013; Kozieł et al., 2021). In this context, it is important to note that plant viruses are specific pathogens that are active only inside the host cell, as they constantly rely on cellular machinery for reproduction (Pogue et al., 2002; Otulak and Garbaczewska, (2014); Kozieł et al., 2021). Thus, plant viruses generally try to keep their hosts alive for as long as possible. This characteristic makes the virus-plant interaction a prolonged one and is often highly dependent on the internal pathways of the host. During stress associated with the presence of biotic/abiotic stressors reactive oxygen species (ROS) are produced. ROS directed plant reaction and are used as signal transduction molecules that control different reaction pathways. Besides biochemical production during stress metabolism ROS are also generated by NADPH oxidases (also named respiratory burst oxidase homologs, RBOHs), peroxidases and other oxidases types (Yadav, 2010). Therefore, final level of ROS molecules could be highly dependent on involvement of many factors. Because of that, plants developed complexed ROS control system based on enzymes (like glutathione reductase-GR, glutathione S-transferase-GST, glutathione peroxidase-GPX) and scavenging non-enzymatic hydrophilic molecules like ascorbate (AsA) and glutathione (GSH) (Noctor and Foyer, 1998). Currently, we know that many (groups of) elements have established themselves as important in the defense of plants against pathogens and plant viruses such as antioxidants, lipids, ROS, jasmonic acid (JA), salicylic acid (SA), and many more (Hernández et al., 2016; Gullner et al., 2017; Koch et al., 2017; Yang et al., 2019; Ding and Ding, 2020; Van Butselaar and Van den Ackerveken, 2020). Among these elements is also glutathione (GSH), which plays a very interesting multifunctional role. As an antioxidant, glutathione enables precise direct or indirect control of ROS (such as singlet oxygen, superoxide anions, hydrogen peroxide, etc.), which often accumulate/produce at high levels during biotic stress, thereby reducing damage to the cells (Hernández et al., 2016; Gullner et al., 2017). This protection is crucial because ROS are not only engaged in signal transduction, but also can oxidize lipids, inhibit enzymes, inactivate biomolecules, and damage proteins, RNA, and DNA, causing a critical level of cell damage. GSH also activates defense pathways against pathogens by mediating between ROS, SA, JA, and ethylene (Alquéres et al., 2013; Han et al., 2013; Ghanta et al., 2014; Cheng et al., 2015; Hernández et al., 2016; Gullner et al., 2017; Künstler et al., 2019a, b; Zechmann, 2020). Moreover, many authors emphasize the high mobility of glutathione; which is systemically transported and serves as a storage form of reduced sulfur, which can be remobilized when needed by plants (Otulak-Kozieł et al., 2022a). Thus, GSH plays the role of a mediator in crucial cellular processes, such as cell cycle progression and programmed cell death (Diaz-Vivancos et al., 2010).

**Figure 1:**
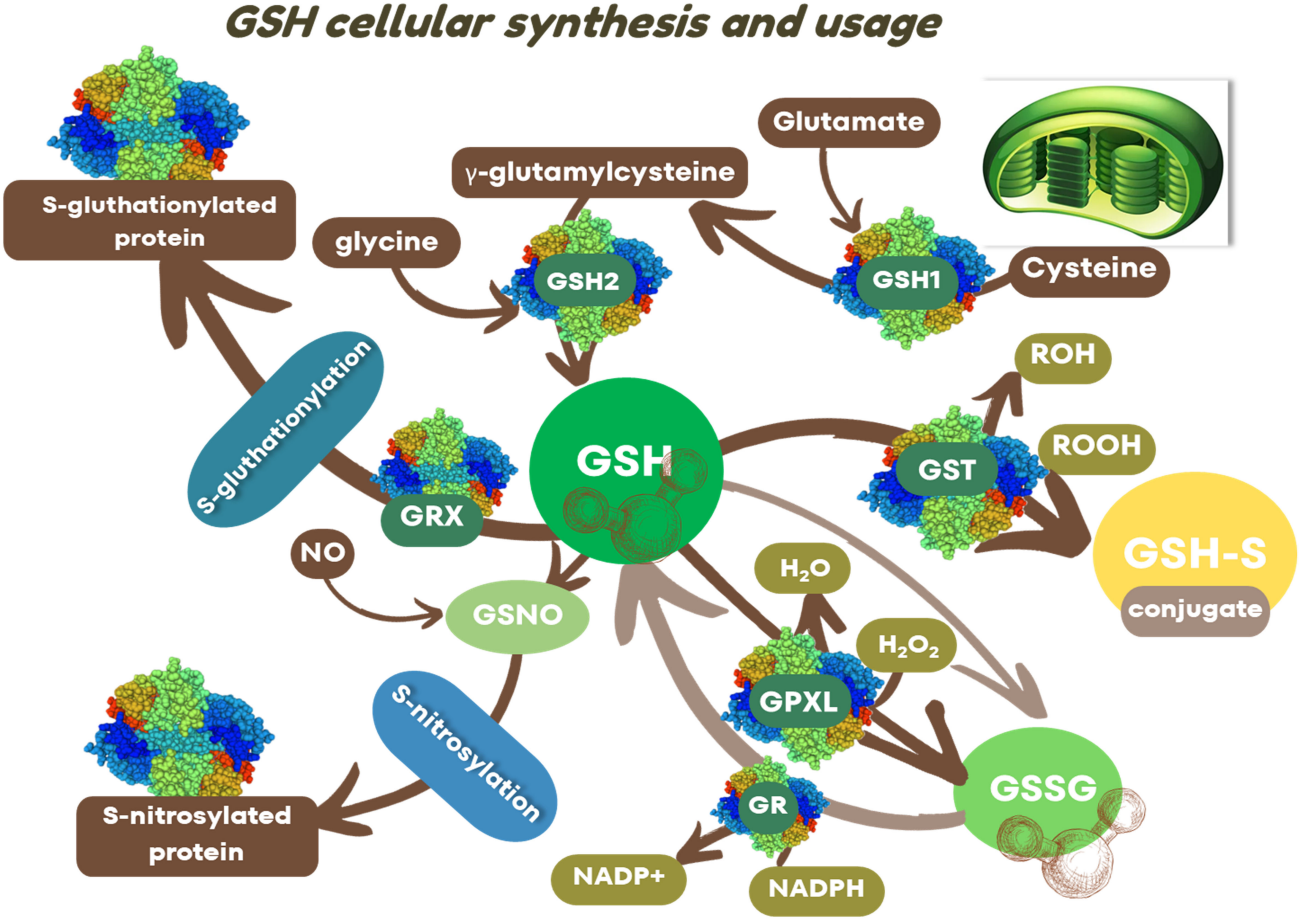
GSH cellular synthesis and usage. γ-glutamylcysteine synthetase (GSH1) active in plastids; GSH synthetase (GSH2) active in plastids and cytoplasm; reduced glutathione form (GSH); oxidized glutathione form (GSSG); glutathione-S-transferase (GST); glutathione peroxidase-like (GPXL), glutaredoxin (GRX); glutathione reductase (GR); *S*-nitrosoglutathione (GSNO).

The GSH itself is created from amino acids, including glutamate, L-cysteine, and glycine, through two ATP-dependent enzymatic reactions mediated by γ-glutamylcysteine synthetase (γ-ECS or also known as GSH1) and GSH synthetase (GS or also named GSH2) (Noctor et al., 2002, 2012; Koramutla et al., 2021). The first and rate-controlling step, catalyzed by γ-ECS, produces γ-glutamylcysteine (γ-EC) from the amino acids L-glutamate and L-cysteine. In the second step, GSH synthetase adds glycine to γ-glutamylcysteine (γ-EC) to produce GSH (**Figure 1**). The reaction catalyzed by γ-ECS/GSH1 is considered the rate-controlling step of GSH synthesis, and the activity of this enzyme is regulated by cellular levels of cysteine and glutamic acid, and feedback inhibition by γ-EC and GSH (Hernández et al., 2015; Koramutla et al., 2021).

GSH1 (EC 6.3.2.2) is exclusively present in plastids, while GSH2 (EC 6.3.2.2) has dual localization in plastids and the cytosol, both encoded by a single gene (Wachter et al., 2005). According to the subcellular localization of GSH1, the GSH synthesis initiates in the plastids, but the predominant transcript, especially in the case of multiple GSH2 transcript populations, encodes a cytosolic GSH2, suggesting the second step occurs in the cytosol (Noctor et al., 2012). After synthesis, GSH can actively move to other cellular compartments, predominantly in its reduced or conjugated forms (Noctor et al., 2012). The reduced GSH form can readily transform into its oxidized form, GSSG, in various biochemical reactions. The cellular homeostasis between the GSH and GSSG ratio controls the cell’s redox level, maintained by reactions performed by glutathione reductases (GR) and glutathione peroxidases (GPX) (Mahmood et al., 2010). The GRs (EC 1.8.1.7) are integral to plant antioxidant defense systems against pathogens, participating in both enzymatic and nonenzymatic oxidation-reduction processes of the cell (Clarke et al., 2022). GRs depend on NADPH levels to transform GSSG to GSH, and through it, they maintain a high ratio of GSH/GSSG in the cell and contribute to the response against plant viruses (Otulak-Kozieł et al., 2023). The plant glutathione peroxidase-like enzymes (GPXL) family consists of multiple isoenzymes with distinct subcellular locations, exhibiting different tissue-specific expression patterns and involvement in various types of stress. Plant GPXLs, containing cysteine in their active site domain, may have dual roles, acting as glutathione peroxidase and thioredoxin peroxidase functions (Bela et al., 2015). The thiol-dependent activities of plant GPXL isoenzymes indicate their role in detoxifying H_2_O_2_ and organic hydroperoxides, as well as their involvement in regulating cellular redox homeostasis by maintaining thiol/disulfide or NADPH/NADP+ ratios (Bela et al., 2015). In this context, GPXL can modulate the levels of NADPH needed for GR activity in the recreation of GSH from GSSG.

Deficiency in the activity of either GSH1 or GSH2 impairs GSH production, negatively impacting plant growth and development. On the other hand, overproduction of glutathione in tobacco mutants, as reported by Künstler et al. (2019a), enhances resistance to tobacco mosaic virus (TMV) infections. Moreover, several factors including the concentrations of cysteine and glycine, the availability of ATP, photosynthetically active photon flux, and enzymes that consume GSH, also regulate GSH biosynthesis (Ogawa et al., 2004; Noctor et al., 2012). Many of these factors undergo changes during viral infections and may influence the redox state of the cell. Once produced, GSH can undergo conjugation with toxic substances (Noctor et al., 2012; Shu et al., 2016) or participate in the modulation of viral infection (Otulak-Kozieł et al., 2022a) through the action of glutathione S-transferase-GST (EC 2.5.1.18) (**Figure 1**). GSH can also serve as a substrate for S-glutathionylation of proteins in the presence of small redox enzyme glutaredoxins (GRX), which also utilizes GSH as a cofactor (**Figure 1**). Additionally, GSH can react with the NO free radical to produce GSNO, which nitrosylates target proteins. The role of S-glutathionylation or S-nitrosylation in plant-virus interaction is not well understood. Sarkar et al. (2011) suggested the involvement of GSNO in the compatible interaction of mesta yellow vein mosaic virus (MeYVMV) with *Hibiscus cannabinus*. In the case of GSTs, these multifunctional and essential enzymes are involved in many processes, such as detoxification, signaling, redox homeostasis, plant metabolism, growth regulation, and adaptation to biotic and abiotic stress (Chronopopulou et al., 2017). GSTs catalyze the conjugation of GSH to various hydrophobic compounds and also perform noncatalytic functions as transporters (Chronopopulou et al., 2017). They also act as signaling markers for infection by various pathogens (Dixon and Edwards, 2010; Sabetta et al., 2017). Deep sequencing investigations have revealed that the glutathione cycle and the expression profiles of GST are regulated by various plant-virus interactions involving *Tobamovirus* (Li et al., 2017), *Geminivirus* (Góngora-Castillo et al., 2012), and *Tenuivirus* (Sun et al., 2016).

A relatively low number of studies have focused on the importance of the glutathione cycle and glutathione-associated enzymes in plant cell responses, both compatible and incompatible, or their potential role in developing resistance to plant viruses. The complete array of plant virus-associated elements involved in the glutathione cycle remains unknown. Hence, this review presents the current understanding of the role of glutathione and glutathione-associated enzymes in the susceptible and resistant responses of plants to viruses. Additionally, it seeks to summarize potential avenues for future research, exploring various aspects of plant-pathogen interactions.

## 2 Role of glutathione cycle in regulation of resistant host-plant virus interaction

**Figure 2:**
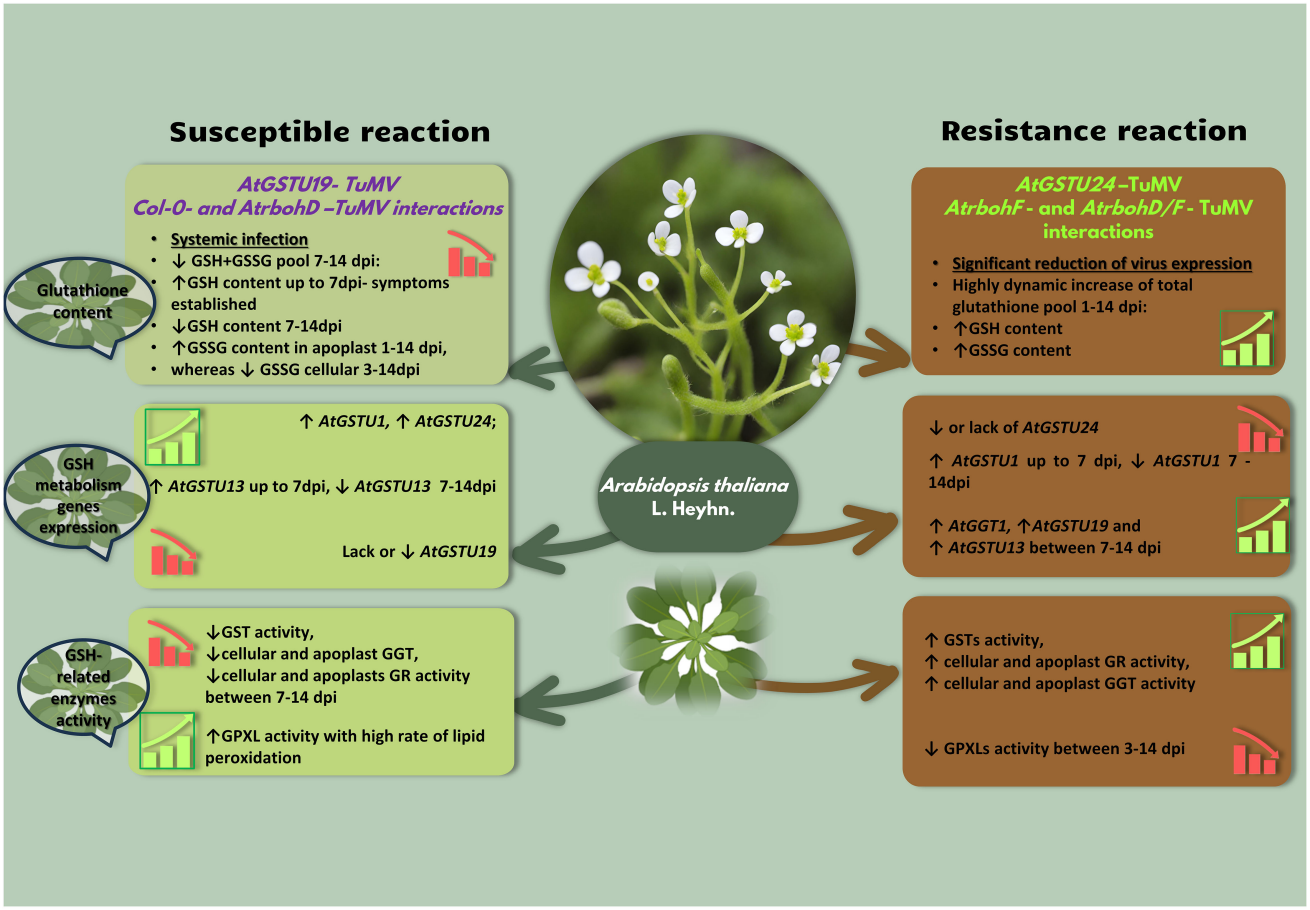
Glutathione content changes, tendency in selected glutathione metabolism- related genes expression and glutathione-associated enzymes activity in susceptible (left, *AtGSTU19*-TuMV, *AtrbohD*-TuMV interaction) as well as resistance (right, *AtGSTU24*-TuMV, *AtrbohF*-TuMV, *AtrbohD/F*-TuMV interaction) *Arabidopsis thaliana* mutants- TuMV reaction. γ-glutamyl transferase (GGT), glutathione peroxidase-like (GPXL), glutathione S-transferase (GST),glutathione S-transferase tau-class (GSTU), reduced glutathione form (GSH), oxidized glutathione form (GSSG), glutathione reductase (GR), ↑-activation or up-regulation, ↓- decrease or down-regulation.

The resistance and tolerance of plants against plant viruses is directly connected with the controlled generation of ROS during virus recognition. This process facilitates signal transduction to inform the plant about infection and enables the initiation of a well-directed defense response (in resistance) or partially directed (in tolerance). Therefore, maintaining a precisely controlled level of ROS is crucial, as overproduction could disrupt plant defense/tolerance responses and lead to direct and serious damage to cells. Elements crucial for ROS control, cell protection, and known antiviral responses include the glutathione cycle, especially the levels of GSH and GSSG forms, along with glutathione-associated enzymes such as glutathione transferases (GSTs), glutathione reductases (GRs), and glutathione peroxidases (GPXs).The involvement of glutathione or glutathione treatment in resistance reactions to plant viruses has been reported for various virus types, including potato virus Y-PVY (Otulak-Kozieł et al., 2022a) on different cultivar of potato, tobacco mosaic virus*—*TMV on tobacco (Gullner et al., 1999; Király et al., 2012; Künstler et al., 2019a, b; Zhu et al., 2021), turnip mosaic virus-TuMV (Otulak-Kozieł et al., 2022b, 2023) on Arabidopsis and obuda pepper virus*-* pepper interaction (Kalapos et al., 2021). Generally, it is suggested that elevated glutathione or its external supplementation improves resistance or tolerance against plant viruses. Gullner et al. (1999) reported that the use of the cysteine precursor L-2-oxo-thazidine-carboxylic acid (OTC also known as GSH activator) on tobacco leaf discs resulted in the accumulation of glutathione and a significant reduction in TMV levels. A similar situation was reported during direct treatment by use of sulfur which inhibited the development of symptoms and limited virus levels in zucchini yellow mosaic virus (ZYMV)-infected pumpkin through an artificial increase in glutathione (Zechmann et al., 2005; Zechmann et al., 2007). Király et al. (2012) indicated that TMV-resistant tobacco plants with adequate sulfate availability showed fewer necrotic symptoms compared to those with a sulfate deficiency. These authors also postulated that virus resistance correlated with an elevated content of glutathione and Cys and the induction of glutathione. Furthermore, they observed that elevated levels of subcellular GSH in interspecific tobacco hybrid plants (*Nicotiana edwardsonii* var. Columbia, NEC) in response to TMV and TNV infection suggest that, in addition to SA, GSH may also contribute to the elevated virus resistance of NEC (Király et al., 2024). On the other hand the increased tolerance reaction against TMV was also confirmed by GSH and OTC treatment of tobacco GSH biosynthesis genes *NbECS* and *NbGS* mutants (Zhu et al., 2021). The results of Clemente-Moreno et al. (2010, 2013) indicated that pea and peach plants treated with OTC characterized tolerant/partially resistant response against plum pox virus (PPV) with lower level of symptoms occurrence. Moreover, after OTC treatment Clemente-Moreno et al. (2013) reported increased plant growth, increased protection to the photosynthetic machinery and the metabolism of chloroplast in PPV-infected in case of peach plants. Clemente-Moreno et al. (2013) suggested that this could be an effect of induction of non-expressor of pathogenesis-related genes 1 (*NPR1*) by OTC treatment. This directly indicated increased tolerance to virus infection stress could be an effect of co-involvement of GSH and *NPR1* which was observed in tolerant interaction of tobacco with other types of pathogens (Ghanta et al., 2011). The GSH-related modulation of virus infection was also reported in the case of tolerant pumpkin and ZYMV (Zechmann and Müller, 2008). The exact mechanism of modulation of tolerance by GSH is still not entirely known, although Zhu et al. (2021) suggested that GSH could cooperate with SA in modulation of that process in case TMV in tobacco (*Nicotiana tabacum*) and in constitutive GSH synthesis during potato virus X (PVX) accumulation control in *Nicotiana benthamiana* (De et al., 2018; Künstler et al., 2019b). However, glutathione levels are not only important during external induction/delivery but also during natural internal production during viral infection. Otulak-Kozieł et al. (2022a) and Otulak-Kozieł et al. (2022b; 2023) detected modulation of glutathione levels during investigations of infections caused by PVY^NTN^ and TuMV on susceptible and resistant (with hypersensitive response or hypersensitive-like, HR or HR-like) respectively on potato and *rbohF* and *rbohD/f* mutants of Arabidopsis (**Figure 2**). Resistant potato plants infected by PVY^NTN^ exhibited a dynamic increase in the content of glutathione during both resistance and, to some extent, susceptible reactions. However, the increase of glutathione (GSH+GSSG and separate GSH and GSSG forms) during HR was more dynamic and stable. This increase correlated with a significant reduction in the amount and expression of PVY^NTN^ and the induction of the HR response. A similar pattern of stable increase in levels of GSH and GSSG was observed in Arabidopsis mutant plants with increased resistance (*rbohF*) and HR-like reaction (*rbohD/F*) infected by TuMV (Otulak-Kozieł et al., 2023), which was also associated with decreased expression of the virus. Singh et al. (2020) reported that resistant cultivars of *Vigna mungo* inoculated with yellow mosaic virus (YMV) also showed an induction of glutathione production, suggesting that plants with viral resistance can potentially elevate the production of glutathione during infection. Fodor et al. (1997) indicated that resistant tobacco Xanthi, in reaction to TMV, exhibited an elevation in GSH, corresponding to results with PVY^NTN^, YMV, and TuMV. However, GSSG levels were slightly decreased in leaves after TMV inoculation, which differed from the observations in resistant potatoes infected by PVY^NTN^. Király et al. (2002) and Künstler et al. (2019b) explained that higher GSSG levels indicated the importance of glutathione in the restoration of TMV resistance, suggesting the suppression of oxidative stress HR in virus-infected cells and downstream defense responses. Otulak-Kozieł et al. (2022a; 2022b; 2023) also reported changes in cellular levels of glutathione content. PVY^NTN^ and TuMV infections significantly elevated the glutathione content in cells of resistant potato and Arabidopsis plants and their mobility to specific cell components. Ultrastructural distribution of glutathione demonstrated by Otulak-Kozieł et al. (2022a; 2022b; 2023) in resistant plants showed that glutathione was mostly deposited in the chloroplast, cytoplasm, and nucleus during PVY^NTN^ and TuMV infections. However, both interactions differed in the case of mitochondria, where in resistant plants against PVY^NTN^, deposition generally remained unchanged, while Arabidopsis plants resistant to TuMV exhibited induced deposition in this organelle. Similar results were reported by Höller et al. (2010) and Zechmann (2020) in resistant tobacco interactions during TMV infection. As postulated by Clemente-Moreno et al. (2015), ROS accumulation is a common feature in plant virus infection. Therefore, not only increased production but also active redistribution of glutathione during the resistant reaction could actively protect vital organelles during infection. Elevated glutathione concentration in the chloroplast is also an important factor for ROS control and symptom development. The breakdown of the oxidative system in the chloroplast is often correlated with necrotic alterations. In the case of mitochondria, Király et al. (2012) indicated that, during incompatible TMV tobacco infection, glutathione depletion induced in the mitochondria correlated with the induction of necrotic lesions in hypersensitive responses. Data from TuMV and TMV suggest that deposition in mitochondria could vary in specific interactions with the host. Nevertheless, Zechmann (2020) and Otulak-Kozieł et al. (2022a) suggested that glutathione plays a very important role in specific cell compartments, activating plant defense and contributing to the development of resistance. Data presented in the case of TuMV infection in Arabidopsis suggest that not only the cell interior but also the apoplast could be a site of modulation of glutathione levels important for resistance (Otulak-Kozieł et al., 2023). The resistant mutants *rbohF* and *rbohD/F* of Arabidopsis exhibited the induction of GSH form deposition and summary glutathione (GSH+GSSG pool) changed the activity of apoplastic GGT (γ-glutamyl transferase) in the apoplast, with the active rerouting of GSSG from the cell wall to the symplast during TuMV infection (**Figure 2**). This movement enables an increased pool of GSSG in the cell for potential use by specific glutathione enzymes like GST, emphasizing the importance of glutathione-associated enzymes as key molecules in the resistant response. In the context of resistance to PVY^NTN^ infection, potato cv. Neptun showed an increased expression of glutathione transferase *StGSTF2* and a general activity of GST, corresponding with an increase in the GSSG form and indicating involvement in the resistance reaction. So increased levels of GSSG in cells that differed from the data reported by Fodor et al. (1997), were the result of a global increase in GST activity in resistant plants. Works by Gullner et al. (1995) and Wu et al. (2013) on sugarcane mosaic virus (ScMV) reported a significant increase in GST activity in resistant sorghum cultivars. Moreover, the importance of GST was also postulated by Chronopopulou et al. (2017), not only as enzymes involved in detoxification and ROS homeostasis but also as signaling molecules and adaptors in biotic stress (Chronopopulou et al., 2017; Gallé et al., 2019, 2021). Additionally, Fodor et al. (1997), indicated that GST plays a pivotal function in controlling HR and necrotization during plant-virus interaction. The importance of GST in resistance as suggested by Otulak-Kozieł et al. (2022a) and Fodor et al. (1997) was confirmed during the investigation of resistant tobacco infected by TMV (Király et al., 2012). During TMV investigations, Király et al. (2012) observed the induction of *NtGSTU1* (from the *tau* group) expression between 3 and 6 h after virus inoculation, which manifested as enhanced HR, causing a reduction in TMV replication in plants with sufficient sulfate. Transcriptomic analyses revealed that the GST expression profile can be differentially regulated in plant-virus interactions. Generally, most GSTs are upregulated rather than downregulated during the resistance reaction, as confirmed in pepper leaves infected with *Obuda pepper virus*—ObPV (Kalapos et al., 2021), rice stripe virus-RSV during infection in *Arabidopsis thaliana* (Sun et al., 2016), *Beta vulgaris* and beet necrotic yellow vein virus-BNYVV interactions (Decroës et al., 2022), and the response of watermelon to cucumber green mottle mosaic virus*-*CGMMV (Li et al., 2017). Furthermore, the expression of specific GST genes was significantly activated in the presence of BNYVV and rice tungro spherical virus (RTSV) in resistance reactions (Larson et al., 2008; Satoh et al., 2013). Satoh et al. (2013) also postulated that rice plants’ resistance to RTSV infection induced not only GST but also the expression of genes encoding GRX, suggesting that s-gluthationylation, with the engagement of GSH, could be important in the resistance reaction. However, depletion of specific GST was shown to influence the induction of a resistant reaction. Rodriguez-Peña et al. (2021) showed that *GSTU4* downregulation caused a significant reduction in the accumulation of barley mosaic virus (BMV) and PVX in a specific host. A study exploring the response of *Atgstu19* and *Atgstu24* mutants to TuMV infection showed significant differences in specific *AtGSTU* gene expression, virus concentration, ultrastructural alterations, glutathione content, and glutathione transferase and reductase activities compared with Col-0 (wild-type) and mock-inoculated plants (Otulak-Kozieł et al., 2022b). Authors reported that *Atgstu24* mutants had a resistance-like reaction to TuMV (with a high decrease in virus gene expression and movement) compared to susceptible Col-0 plants, suggesting that *GSTU24* may suppress plant resistance. Moreover, this mutant had upregulated expression of *GSTU19* and *GSTU13* highly correlated with virus limitation in the resistance-like reaction (Otulak-Kozieł et al., 2022b). Moreover, resistant *Atgstu24* mutants also characterized the upregulated activity of GR. Similarly, Otulak-Kozieł et al. (2023) reported that resistant *rbohF* and *rbohD/F* mutants infected by TuMV had increased activity of GST and GR, strongly downregulated GPXL, and highly reduced levels of lipid peroxidation. The same situation was also reported by Kalapos et al. (2021), showing high suppression of GPXL based on the results of transcriptome profiling during ObPV–*C. annuum* in HR. On the other hand, the more tolerant of tobacco plants to pepper mild mottle virus (PMMoV-I) infection characterized decreased activity GR whereas OTC treated tolerant pea characterized GR upregulation (Clemente-Moreno et al., 2010; Hakmaoui et al., 2012).

## 3 Role of glutathione cycle in regulation of susceptible host-plant virus interaction

In contrast to resistance, in susceptible plants, the generation of ROS is not well or properly controlled due to changes induced by plant virus infection in the host cell, particularly in the modulation of glutathione. In this case, the modulation is closely associated with the levels of GSH and GSSG forms. As reported in the cases of PVY^NTN^ and TuMV, the GSH form and total glutathione levels could be upregulated in susceptible plants until the point of symptom occurrence and then highly depleted (Otulak-Kozieł et al., 2022b, 2023). In contrast to this, the GSSG form concentration decreased during infections caused by, for example, PVY^NTN^ and TuMV (Otulak-Kozieł et al., 2022a, b). The depletion of glutathione content in susceptible reactions was also reported by Hakmaoui et al. (2012); Singh et al. (2020), and Király et al. (2012) for PMMoV in tobacco, YMV in black gram, and TMV in tobacco respectively. This data, along with reports by Hernández et al. (2017), indicates that susceptible plants, to some extent, are able to control ROS production and delay symptom development. However, without launching a resistant response, the protective potential of the GSH form (via upregulation of synthesis and specific activity of glutathione-associated enzymes) is limited, and the ability to regain GSH from GSSG is mitigated with less presence of the GSSG form. The mobility of glutathione also has an impact on in-cell and in-apoplast relocation in susceptible plants. In susceptible potatoes infected at later stages of PVY^NTN^ infection,the localization of total glutathione decreased, even to nonstatistically significant levels in the cytoplasm and chloroplast. The mutants *AtGSTU19* infected by TuMV had decreased content of glutathione in mitochondria, cytoplasm, nucleus, vacuole, and chloroplast. A similar situation was observed in *rbohD* mutants of Arabidopsis, also exhibiting decreased apoplastic localization (Otulak-Kozieł et al., 2023). During susceptible interactions, not only is glutathione content and distribution changed but there are also changes in the expression of genes encoding specific enzyme changes (**Figure 2**). In the context of transcriptomic analyses, it is generally observed that GST expression is downregulated in susceptible interactions (Sun et al., 2016; Li et al., 2017; Kalapos et al., 2021; Decroës et al., 2022). However, in specific plant-virus interactions, certain GSTs may be induced. For instance, during the infection of susceptible *A. thaliana* by cauliflower mosaic virus (CaMV), systemic induction of *GST1* was associated with increased virus titers and the development of disease symptoms (Love et al., 2005). Pavan Kumar et al. (2017) also reported the accumulation of some GST proteins in systemically infected leaves of soybeans susceptible to mungbean yellow mosaic India virus (MYMIV) and mungbean yellow mosaic virus (MYMV). Additionally, Zhang et al. (2022) documented that *Glycine max GSTU13* was associated with the development of symptoms induced by soybean mosaic virus (SMV) at both transcriptional and protein levels. Furthermore, the works of Chen et al. (2013) and Skopelitou et al. (2015) demonstrated the upregulation of *NbGSTU4* and *GSTU10-10* during infections caused by bamboo mosaic virus (BaMV) and SMV on susceptible hosts. Chen et al. (2013) also postulated that the NbGSTU4 protein has the ability to bind to the UTR region of (+) s virus RNA, leading to effective replication in susceptible hosts. In tomato cultivars tolerant to tomato leaf curl New Delhi virus (ToLCNDV), Sharma et al. (2021) observed significant upregulation of *SlGR3*, *SlGST44*, and *SlGST96* during virus infection and different hormone treatments in the tolerant cultivar. Moreover, the virus-induced gene silencing of *SlGR3* turned the tolerant cultivar into a susceptible one. Méndez-López et al. (2023), in their investigation of pepino mosaic virus (PepMV) on susceptible tomatoes, showed that *SlGSTU38* acted as a susceptibility factor and outlined the dual role of the proviral SlGSTU38 protein. It was suggested that the SlGSTU38 protein interacted with PepMV capsid protein and played a role in delaying virus infection by engaging in or disturbing redox homeostasis. Otulak-Kozieł et al. (2022b) also speculated that similar viral-host protein interactions could occur in the case of GSTU19 and GSTU24 proteins during TuMV infection in different Arabidopsis mutants in various types of interactions. Based on these studies, it is suggested that not only the expression of specific GSTs but also direct interactions between GST proteins and the virus may be necessary to overcome defense mechanisms in susceptible plants. This observation aligns well with the crucial ability of viruses to interact with host proteins (for example in the *Potyviridae* family), leading to the induction and support of virus infection in different hosts (Chen et al., 2013; Yang et al., 2021). In the analysis of the *Atgstu24/Atgstu19-*TuMV pathosystem, it was found that the mutation of specific GSTs also had an effect on generating increased susceptibility in the interaction with the virus. Plants with the *Atgstu19* mutation exhibited increased susceptibility compared to the already susceptible Col-0 plants, which was associated with elevated levels of TuMV expression. Additionally, in Col-0 plants, there was a general decrease in *Atgstu19* expression after 7dpi, indicating that the elimination or limitation of *Atgstu19* expression was crucial for the susceptibility interaction with TuMV. The same study also showed that *AtGSTU1* and *AtGTU24* genes were significantly altered and involved in susceptibility. Not only gene expression but also GST enzymatic activity is modulated during virus infection. Fodor et al. (1997) observed a decrease in the activity of some antioxidant enzymes, especially GST and GR, in susceptible tobacco infected by TMV. Gullner et al. (1995) and Wu et al. (2013) found decreased GST activity in susceptible sorghum cultivars during interaction with ScMV. Similar changes in reduced activity of GST and GR were reported in susceptible mutants infected with TuMV (Otulak-Kozieł et al., 2022b, 2023) after 7dpi which was associated with increased activity of GPXL in *rbohD* mutants (Otulak-Kozieł et al., 2023). The reduction of cellular or chloroplast GR activity was also reported in infections on compatible hosts caused by various viruses such as cocksfoot mottle virus (CfMV) on *Dactylis glomerata*, cucumber mosaic virus (CMV) on tomato, PPV, and prune necrotic ringspot virus (PNRSV) on apricot plants, as well as white clover mosaic virus (WCIMV) on bean plants (Li and Burritt, 2003; Amari et al., 2007; Song et al., 2009; Clemente-Moreno et al., 2015). The involvement of GR in the reduction of glutathione disulfide (GSSG) to two molecules of GSH makes this enzyme crucial for maintaining the glutathione redox potential. Therefore, the reduction of GR activity coupled with an increase in GPXL as reported in the case of YMV or TuMV (Singh et al., 2020; Otulak-Kozieł et al., 2023) creates a situation of poorly controlled and imbalanced redox hemostasis. This imbalance leads to damage of cell components such as uncontrolled lipid peroxidation and blocks the possibilities of proper initiation of defense response at the right place and time to effectively stop the infection.

## 4 Future prospects

In recent years, the importance of controlling redox homeostasis, particularly through glutathione, has been increasingly recognized as crucial for inducing a resistant response. However, our understanding of the exact mechanisms and the significance of glutathione, as well as the involvement of specific glutathione-associated enzymes, remains limited in the context of plant-virus interactions. This limitation is particularly evident in understanding the roles of GRX, GSNO, S-glutatylation, and S-nitrosylation processes. This is mainly due to the fact that research has traditionally focused on well-known stress molecules like SA or JA, or simply measured the activity of selected redox enzymes. The new findings highlight the importance of glutathione mobility within the cell and the direct interaction of glutathione-associated enzymes with viral factors or vRNA essential for the full-fledged development or initiation of viral infection (particularly through interactions with UTR sites in vRNA). This opens up a unique and promising new field of research. To advance our understanding, investigating the relocation of glutathione, both between different cell regions and its dynamic changes, along with gathering transcriptional data specifically focused on glutathione metabolism, will be crucial. Additionally, exploring the direct interactome of glutathione-associated proteins can help greatly in the identification of crucial elements in host-plant virus interplay. The generation of mutants for selected genes based on transcriptomic data, using advanced techniques such as CRISPR/Cas9, will further open new horizons for developing resistance to viruses or other multifactorial stresses.

## Author contributions

EK: Writing – review & editing, Writing – original draft, Project administration, Funding acquisition, Formal analysis. KO-K: Writing – review & editing, Writing – original draft, Supervision, Formal analysis, Conceptualization. PR: Writing – review & editing, Visualization, Software.
